# Research on outpatient capacity planning combining lean thinking and integer linear programming

**DOI:** 10.1186/s12911-023-02106-6

**Published:** 2023-02-13

**Authors:** Li hua, Mu Dongmei, Yang Xinyu, Zhang Xinyue, Wang Shutong, Wang Dongxuan, Peng Hao, Wang Ying

**Affiliations:** 1 Abdominal Ultrasound Department, Diagnostic Ultrasound Center, First Hospital of Jilin University, Changchun, Jilin China; 2 Department of Clinical Research, First Hospital of Jilin University, Changchun, Jilin China; 3 School of Public Health, Jilin University, Changchun, Jilin China

**Keywords:** Outpatient capacity planning, Lean thinking, Integer programming, Clinical pathway, Cplex

## Abstract

**Background:**

The size and cost of outpatient capacity directly affect the operational efficiency of a whole hospital. Many scholars have faced the study of outpatient capacity planning from an operations management perspective.

**Objective:**

The outpatient service is refined, and the quantity allocation problem of each type of outpatient service is modeled as an integer linear programming problem. Thus, doctors’ work efficiency can be improved, patients’ waiting time can be effectively reduced, and patients can be provided with more satisfactory medical services.

**Methods:**

Outpatient service is divided into examination and diagnosis service according to lean thinking. CPLEX is used to solve the integer linear programming problem of outpatient service allocation, and the maximum working time is minimized by constraint solution.

**Results:**

A variety of values are taken for the relevant parameters of the outpatient service, using CPLEX to obtain the minimum and maximum working time corresponding to each situation. Compared with no refinement stratification, the work efficiency of senior doctors has increased by an average of 25%. In comparison, the patient flow of associate senior doctors has increased by an average of 50%.

**Conclusion:**

In this paper, the method of outpatient capacity planning improves the work efficiency of senior doctors and provides outpatient services for more patients in need; At the same time, it indirectly reduces the waiting time of patients receiving outpatient services from senior doctors. And the patient flow of the associate senior doctors is improved, which helps to improve doctors’ technical level and solve the problem of shortage of medical resources.

## Introduction

Outpatient service is one of the key service resources of the medical service system, and it is the earliest place for patients to contact for medical treatment. The efficiency of outpatient resource scheduling directly affects the operation efficiency of follow-up departments and the whole hospital [1]. However, most medical institutions currently face the problems of a large demand for outpatient services but low operation efficiency, long waiting times, and short treatment times for patients [2]. In order to alleviate the contradiction between the supply and demand of outpatient resources, it is urgent to improve the utilization rate of medical resources [3]. How to efficiently schedule and operate limited medical resources and guide patients to seek medical treatment orderly have become the primary task of outpatient scheduling research.

Long waiting time in the outpatient department greatly impacts timely access to medical services and medical experience. Many scholars have studied the problem of long waiting times for patients. Young patients may arrive earlier or later than the appointment time in many cases, and patients' unpunctuality have a negative impact on the normal operation of the appointment arrangement system as planned [4]. It affects the utilization of medical resources and the efficiency of healthcare staff, which increases healthcare costs and reduces the ability of clinics to serve patient populations. Laan et al. proposed a solution of stochastic mixed integer programming based on the fluctuation of patient arrival and the unavailability of doctors to optimize appointment scheduling related to access time [5]. Discrete event simulations are used to evaluate the effectiveness and limitations of the approach, which flexibly allocated 2% of patient flow, resulting in improved clinic performance. Zhu et al. proposed a simulation framework combined with heuristic strategies to improve the performance of the reservation scheduling system given patients' unpunctuality [6]. This method has dramatic advantages in current practice, thus improving the patient capacity of the clinic.

In recent years, the study of outpatient flow scheduling has attracted more and more attention from business and academic circles. Najmuddin et al. developed an optimized scheduling method combined with a discrete event simulation model to reduce further waiting time and increase flow in obstetric outpatients and improved the optimization effect of this method through multiple simulations run [7]. Lenin et al. optimized the appointment model of obstetrics and gynecology clinics and distinguished three different patient types according to the possibilities of each type of patient attending appointments [8]. The best solution resulted in adding a medical assistant and modifying the appointment system to eliminate bottlenecks without sacrificing resource utilization by reducing patient waiting times. Viana et al. proposed a hybrid discrete event simulation optimization model for pregnancy clinics to better plan medical resources and improve patient flow in the outpatient department [9]. This model can effectively deal with uncertain events such as the expansion of catchment areas, the modification of overdue pregnancy guidelines, and women being able to deliver before an appointment.

Doctors' time is precious in medical care, and medical resources are minimal. In order to better play the role of limited resources, many scholars began to use integer programming methods to optimize resource allocation. During the COVID-19 emergency, social distancing was identified as one of the most effective measures to limit the spread of the virus. Caselli et al. proposed an integer linear programming model to determine the optimal layout of outpatient services to reduce the congestion in the waiting room [10]. The experimental results on real hospital data show that this method can reduce congestion by 80% on average. In view of the uncertainty of patients' reservation demand, Aslani et al. proposed the outpatient capacity planning method using cardinality constraints [11]. This method considers the first-visit and the second-visit patients and provides a feasible capacity allocation scheme for all reservation requirements within the allowable range of uncertain appointments. Izady et al. proposed a compact planning scheme for different reservation needs. They proposed a model of reserving free space for more reservation services, thereby reducing patients waiting time [12]. By adjusting the working hours to improve patient flow, overtime costs are minimized under the premise of ensuring the constraints of waiting and appointment access. Santibanez et al. put forward a mixed integer programming model for operating room management, which arranges each specialty’s operation blocks into the operating room [13]. At the same time, considering the time availability of the operating room and the limitation of postoperative resources, the experimental results show that the hospital can handle more cases through different specialty arrangements without increasing postoperative resources.

Lean thought originated from the lean production mode invented by Toyota in Japan. The lean production mode brought advantages in quality and cost to Japanese cars, which then made the automobile industry in other countries unable to rise. The core of lean thinking is to create as much value as possible with less manpower, equipment, time, and space [14]. Healthcare organizations have begun to apply this approach to hospital management [15]. The University of Michigan Health System adopts lean thinking as its unified approach to quality improvement and strives to become a completely lean organization. Many scholars also use lean thinking to improve the management efficiency of the hospital [16]. Improta et al. apply lean thinking to the hospital’s emergency room to increase the flow of patients, improve the process that helps promote the flow of patients in all stages of medical treatment, and eliminate all bottlenecks and activities that produce waste [17]. Mu et al. combined lean thinking to optimize the flow of the obstetric ward, and divided the obstetric ward into an observation ward, cesarean section ward, and natural delivery ward according to lean thinking [18]. The problem of how to allocate the number of wards of each type is modeled as a mixed integer programming problem, which maximizes the patient flow of pregnant women in obstetric hospitals. It increases the patient flow by 19–25% compared to before improvement.

The above methods have been studied on outpatient patient flow and waiting time issues. However, there has yet to be a report on methods of planning outpatient capacity from a more refined perspective. Based on lean thinking, this paper proposes dividing outpatient service into two types: examination service and diagnosis service. Further refinement and classification of outpatient services will make it easier for doctors to manage patients with more centralized distribution, thus indirectly improving their work efficiency. Patients can enjoy more detailed medical services at different stages, thus obtaining more professional medical services in unit time and effectively reducing patient waiting time.

It is a matter of planning how the amount of time allocated for each type of clinic is divided into more detailed sections. Suppose the service time of a certain type of outpatient service is allocated relatively less. In that case, there will be a bottleneck and more patients cannot make an appointment, wasting medical resources. Suppose one type of clinic has a relatively high allocation of service time and the other type has a relatively low allocation of service time. In that case, the patient will be hindered from moving to the next process. In this paper, the outpatient capacity planning problem is modeled as an integer linear programming model where the maximum outpatient working time is minimized. The model is solved by means of an integer linear programming solver. This method has an important guiding role in the planning of outpatient capacity, especially how to plan the working time of different types of outpatient service when the number of patients or doctors on duty changes greatly.

## Methods

### Lean outpatient capacity planning

Famous or senior doctors are scarce resources that are seriously insufficient. Patients often fail to see doctors on time, and doctors work overtime. Because of the natural advantages of their platforms, top hospitals attract patients and service leaders who should have seen doctors in other hospitals or grass-roots hospitals, which is called the siphoning effect in the industry. The siphon effect not only attracts patients from primary hospitals, but also simultaneously "hollows out" senior talents from primary hospitals. Also, due to the siphonage effect, more patients come to the top hospitals. This leads to long patient waiting times, which seriously affects the medical experience.

The worse situation is that there is no appointment with a senior doctor, which also leads to the failure of some incurable diseases to receive the diagnosis and treatment services of senior doctors, seriously affecting the fairness of medical resource allocation. Senior doctors have been engaged in clinical and academic research for many years in the hospital, and have a high level of professional technology and academics. Associate senior doctors generally have been engaged in clinical and academic research for a short time, and need further study in professional technology and academia. They can only be employed as senior doctors after passing the examination. For associate senior doctors, relatively few patients choose them for treatment. Doctors often wait for patients, which leads to a waste of medical resources. More importantly, this situation severely limits the associate senior doctors' learning and accumulation of diagnosis and treatment experience for difficult diseases, which is not conducive to the growth of associate senior doctors.

**Fig. 1:**
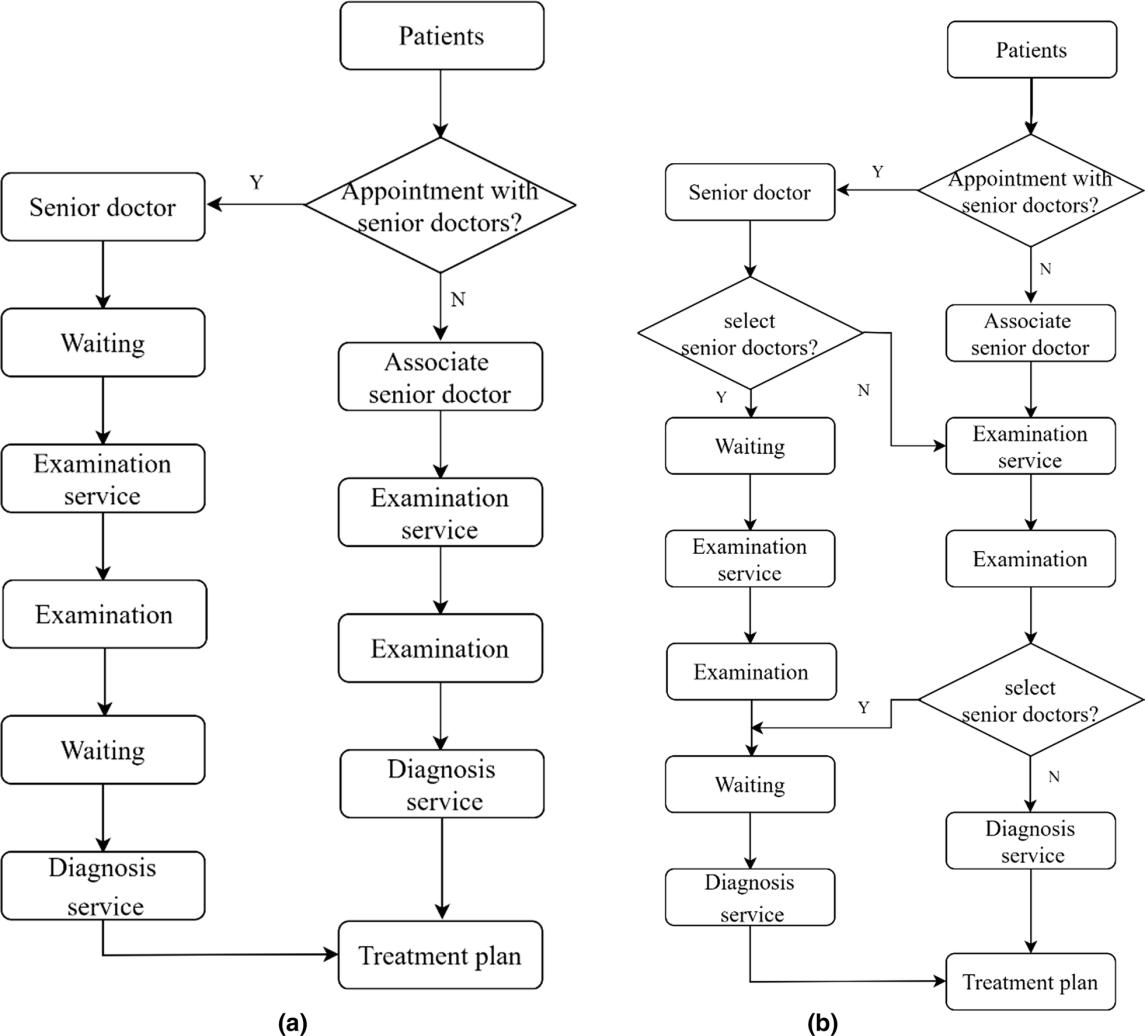
**a** Clinical pathway of outpatient services (Normal). **b** Clinical pathway of outpatient services (Refined)

The general clinical pathway of patients receiving outpatient services from senior doctors is shown in Fig. 1a. Patients line up outside senior doctors' offices during their appointments. When it is the patient’s turn to receive outpatient service, the patient presents the chief complaint in front of the senior doctor, who gives medical advice after understanding the situation. The patient then pays the prescribed fee and has the corresponding medical examination as ordered. After receiving the results of medical examinations, patients return to the doctor’s office and wait in line. When the patient receives outpatient service, the doctor will diagnose the disease according to the examination results and give the corresponding treatment plan.

Patients should communicate with senior doctors at least twice when they receive outpatient services. In some cases, they will have a third or more communication with senior doctors to change their medical orders or ask for treatment plans. Suppose a patient has an appointment with a senior doctor. In that case, the patient needs to wait in line every time he or she receives the services of the senior doctor because the senior doctor is serving other patients. Patients have to wait in line several times to receive outpatient services from senior doctors. For patients, time is relatively tight, with the vast majority of examinations to be completed in the morning or before work. Otherwise, they have to make another doctor's appointment the next day. Usually, patients' waiting time is more than the communication time with senior doctors, and the long waiting time seriously affects patients' rest and emergency treatment, and seriously affects their medical experience. During this period, patients often occupied the time of senior doctors, wasting medical resources and failing to receive more accurate medical services. For senior doctors, when they provide fixed medical services, the time period for their services is not concentrated. Multiple visits to each patient, and senior doctors have to re-master each patient's condition, seriously consuming the energy and time of senior doctors. This has led to more effort and time for senior doctors to provide the same service. What is more, senior doctors are not serving more patients with more time and energy. Instead, fewer patients may be served because of energy consumption and time delays.

The outpatient clinical pathway of the senior doctors mentioned above is the mainstream clinical pathway adopted by the hospital at present. This clinical pathway is mostly a guide in time, without planning for the doctors and space to implement the clinical pathway. On the premise of not changing the clinical pathway, this paper combined lean thinking to plan the implementation of the clinical pathway from the perspective of space and doctors. Based on lean thinking, outpatient services are divided into two types of services in more detail: examination services and diagnosis services. The examination service includes the chief complaint and the first doctor's order. Beyond that, all phases are divided into diagnosis services. In most hospitals, the classification of departments is very fine, and the senior associate doctor is fully competent for patient examination services. According to lean thinking, the examination services of patients who make appointments with senior doctors are assigned to associate senior doctors. In contrast, the diagnosis services are still assigned to senior doctors. The post-planning outpatient service flow is shown in Fig. 1b. Patients wish to receive examination services from non-senior doctors and diagnosis services from senior doctors, see Fig. 1b. Patients will receive examination services from the senior associate doctor first. After completing the appropriate examination services, the patient is returned to the senior doctors for diagnosis services. The procedure for patients who want to receive examination and diagnosis services from senior doctors is represented in Fig. 1a.

Such lean planning has the following advantages: Patients can reduce the waiting time of patients without affecting their medical treatment. Because the number of patients treated by associate senior doctors is small, the medical experience of patients is improved; For senior doctors, energy consumption and working time are reduced so that more energy and time are available to provide services to other patients in need. For the associate senior doctors, the number of patients received has significantly increased, which helps improve the professional level but also helps to solve the problem of difficult medical treatment.

This model is proposed based on many years of research on outpatient service of a class III class A hospital in China. This model has a guiding significance for managing door diagnostic services in all hospitals. This model is still in the theoretical research stage, and further application in practice is the next work to be carried out.

### Integer programming model

This paper’s outpatient scheduling problem is modeled as an integer linear programming model. The constraints of outpatient doctors, examination service time, diagnosis service time, and a number of patients are given below, and then the mathematical programming model of outpatient scheduling is constructed.

Assume that the total number of doctors is *td*, the number of senior doctors is *tds*. Assume that the total number of patients is *tp*, and examination and diagnosis service times are marked as exam_time and diagnosis_time respectively. Two-dimensional data *dpat*[][] is used to represent the appointment relationship between all patients and each doctor. *npp*[] indicates that each doctor can be transferred in or out the number of patients. Transferring in a patient refers to letting the current doctor serve the patient who has made an appointment with the other doctor. Transfer out a patient refers to letting other doctors serve the patient who has made an appointment with the current doctor. The maximum working time of each doctor is set as work_time[], and the maximum working time after scheduling optimization is set as LWT.

Table 1 gives the description of the decision variables and parameter variables related to the mathematical model of this planning. Based on the above constraints, the integer linear programming solver is used to solve, and then the maximum working time of the outpatient department is minimized.

**Table 1:** Description of parameters and decision variables of the problem

Name	Constraint type	Data type	Abbreviation	Description
LWT	Decision	Continuous non-negative	*LWT*	Maximum working time after planning
Npp[]	Decision	Continuous non-negative	*npp*	The number of patients transferred in and out by each doctor after scheduling
Total_doctor	Parameter	Continuous non-negative	*td*	Total number of doctors
Total_patient	Parameter	Continuous non-negative	*tp*	Total number of patients
Doctor_pat[][]	Parameter	Binary	*dpat*	Each patient's appointment relationship with the doctor
Exam_time	Parameter	Continuous non-negative	*et*	Examination service time per patient
Diagnosis_time	Parameter	Continuous non-negative	*dt*	Diagnosis service time per patient
Work_time[]	Parameter	Continuous non-negative	*wt*	Maximum working time per doctor
Senior_doctor	Parameter	Continuous non-negative	*SD*	Number of senior doctors
A-Senior_doctor	Parameter	Continuous non-negative	*ASD*	Number of Associate senior doctors
Sen_doctor_pat	Parameter	Continuous non-negative	*SDP*	All the patients that each doctor has to service
A_Sen_doc_pat	Parameter	Continuous non-negative	*ADP*	All the patients that each associate doctor has to service

The working time of each doctor *i* after scheduling given by the sum of diagnosis, examination, and transfer times should not exceed their limit on working time work_time[*i*]:

$$\mathop \sum \limits_{j = 0}^{tp - 1} \left( {et + dt} \right)*dpat\left[ i \right]\left[ j \right] + et*npp\left[ i \right] \le work\_time\left[ i \right],\;0 \le i < td.$$

For each senior doctor, the working time after scheduling should not exceed $LWT$:

$$\mathop \sum \limits_{j = 0}^{tp - 1} \left( {et + dt} \right)*dpat\left[ i \right]\left[ j \right] + et*npp\left[ i \right] \le LWT,\;0 \le i < tds.$$

For associate senior doctors, the working time after scheduling should not exceed $LWT$:

$$\mathop \sum \limits_{j = 0}^{tp - 1} \left( {et + dt} \right)*dpat\left[ i \right]\left[ j \right] + et*npp\left[ i \right] \le LWT,\;tds \le i < td.$$

For senior doctors, if they have more patients booked than the average number of outpatient visits, they should call out some patients. Otherwise, some patients should be enrolled. The corresponding constraints are as follows:

$$\forall i,\;0 \le i \le tds - 1,npp[i]\left\{ {\begin{array}{*{20}l} { \le 0,\mathop \sum \limits_{i = 0}^{tds - 1} dpat\left[ i \right]\left[ j \right] > tp/td} \hfill \\ { \ge 0,\mathop \sum \limits_{i = 0}^{tds - 1} dpat\left[ i \right]\left[ j \right] \le tp/td} \hfill \\ \end{array} } \right..$$

For associate senior doctors, if the number of patients they have booked is greater than the average number of outpatient reception, patients will not be transferred. Otherwise, some patients should be enrolled. The corresponding constraints are as follows:

$$\forall i,\;td \le i \le td - 1,npp[i]\left\{ {\begin{array}{*{20}l} { \le 0,\mathop \sum \limits_{i = tds}^{td - 1} dpat\left[ i \right]\left[ j \right] > tp/td} \hfill \\ { \ge 0,\mathop \sum \limits_{i = tds}^{td - 1} dpat\left[ i \right]\left[ j \right] \le tp/td} \hfill \\ \end{array} } \right..$$

For the number of patients transferred in and out by all doctors, its sum should remain unchanged. The corresponding constraints are as follows:

$$\mathop \sum \limits_{i = 0}^{td} npp\left[ i \right] = 0.$$

This planning problem is to solve the minimization of the maximum working time of the outpaent department, and its constraints are as follows.

The objective of the considered planning problem is to minimize the maximum working time of the outpatient department, which is formulated as follows:

$${\text{minimize}}\quad {\text{LWT}}{.}$$

## Results

Today, more than 1000 universities and more than 100 leading software companies worldwide choose to use the IBM CPLEX optimizer to help solve planning problems in various industries [19]. The CPLEX optimizer provides flexible, high-performance mathematical programming solutions for integer programming problems and is available in free versions for educational and scientific research.

In this paper, the above outpatient planning problems are coded in CPLEX, and CPLEX is used as a mixed integer programming solver. Our method is proposed based on many years of research and practice on the outpatient services of a class III A Chinese hospital. The data used in our experiments are generated based on the real activity of this hospital. All experiments are run on the hardware of the Windows 10 64-bit operating system. Due to the small scale of the problem and the high efficiency of CPLEX in solving integer programming problems, all test cases are solved within 1 min.

Table 2 shows the minimum of the maximum working time obtained by using CPLEX and the patient information adjusted by senior doctors and associate senior doctors. Detailed parameters corresponding to each test are shown in Table 3. RTS represents the improvement of the work efficiency of senior doctors receiving the same number of patients after planning and before planning; RTDS represents the improvement of the number of patients received by associate senior doctors after planning and before planning. Their calculation formula is as follows. SNPP and ANPP represent the number of patients transferred in or out by senior doctors and associate senior doctors respectively.

$${{{\text{RTS}} = {1} - {\text{LWT}}*{\text{SD}}} \mathord{\left/ {\vphantom {{{\text{RTS}} = {1} - {\text{LWT}}*{\text{SD}}} {\left( {\mathop \sum \limits_{i = 0}^{tds} doctor\_pat\left[ i \right]\left[ j \right]*\left( {et + dt} \right)} \right)}}} \right. \kern-0pt} {\left( {\mathop \sum \limits_{i = 0}^{tds} doctor\_pat\left[ i \right]\left[ j \right]*\left( {et + dt} \right)} \right)}}$$

$${{{\text{RTDS}} = {\text{LWT}}*{\text{ASD}}} \mathord{\left/ {\vphantom {{{\text{RTDS}} = {\text{LWT}}*{\text{ASD}}} {\left( {\mathop \sum \limits_{i = tds + 1}^{td} doctor\_pat\left[ i \right]\left[ j \right]*\left( {et + dt} \right)} \right)}}} \right. \kern-0pt} {\left( {\mathop \sum \limits_{i = tds + 1}^{td} doctor\_pat\left[ i \right]\left[ j \right]*\left( {et + dt} \right)} \right)}} - {1}$$

**Table 2:** Results of planned outpatient capacity

Input parameters					Results			
LWT	ET	DT	SD	ASD	SNPP	ANPP	RTS	RTDS
272	8	2	10	10	− 11	11	0.24	0.51
271	7	3	10	10	− 13	13	0.25	0.51
270	6	4	10	10	− 15	15	0.25	0.50
270	5	5	10	10	− 18	18	0.25	0.50
272	4	6	10	10	− 22	22	0.24	0.51
270	3	7	10	10	− 30	30	0.25	0.50
288	2	8	10	10	− 36	36	0.20	0.60
210	5	5	2	18	− 30	6	0.42	0.17
235	5	5	4	16	− 26	10	0.35	0.31
250	5	5	6	14	− 22	14	0.31	0.39
260	5	5	8	12	− 20	16	0.28	0.44
280	5	5	12	8	− 16	20	0.22	0.56
295	5	5	14	6	− 13	23	0.18	0.64
310	5	5	16	4	− 10	26	0.14	0.72
330	5	5	18	2	− 6	30	0.08	0.83

**Table 3:** Parameters for experiments

Factor	TD	TP	ET	DT	WT	SD	ASD	SDP	ADP
ET	20	540	–	–	360	10	10	36	18
DT	20	540	–	–	360	10	10	36	18
SD	20	540	5	5	360	–	–	36	18
ASD	20	540	5	5	360	–	–	36	18

According to the data obtained from the experimental solution after the planning, the work efficiency of senior doctors after the planning increased by 42% at the highest, 8% at the lowest, and 24% on average. The number of associate senior doctors increased by 83% at the highest level, 16% at the lowest level, and 51% on average. At present, the ratio of senior and associate senior doctors in most class III class A hospitals is 1:1. In this proportion, after using the method proposed in this paper, the work efficiency of senior doctors is up to 25%, and the number of patients of associate senior doctors is up to 50%.

According to the experimental results, senior doctor’s work efficiency has improved, and the number of patients treated by associate senior doctors has increased significantly. This is due to the fact that the associate senior doctors are assigned to take care of the examination service of patients who make appointments with senior doctors. Senior doctors can save more time when serving the same number of patients. At the same time, it also effectively reduces patients’ waiting time in the senior doctor. For associate senior doctors, the opportunity to contact more patients every day is increased, which is conducive to the cultivation of professional skills.

**Fig. 2:**
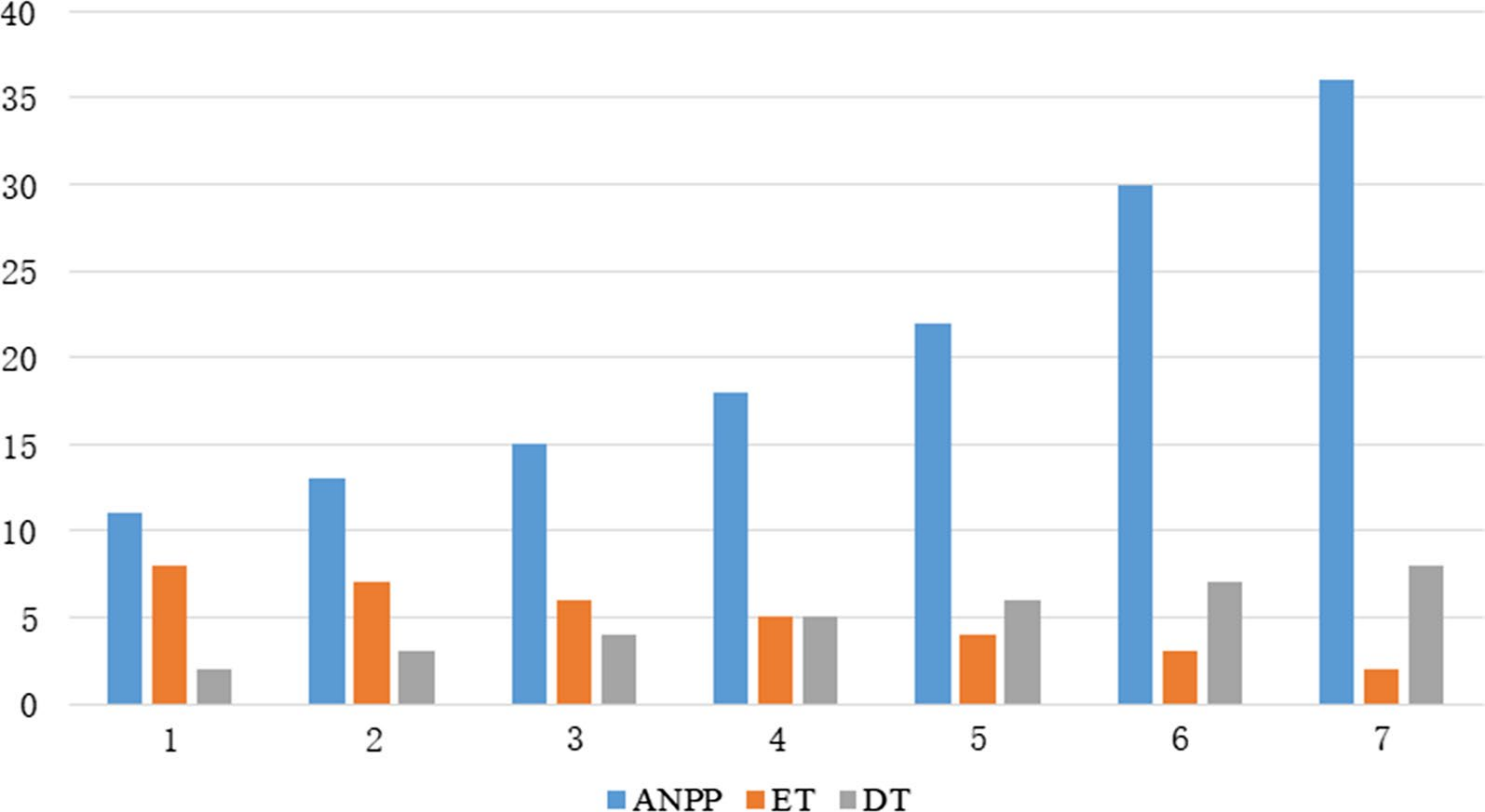
Patients of ET and DT

**Fig. 3:**
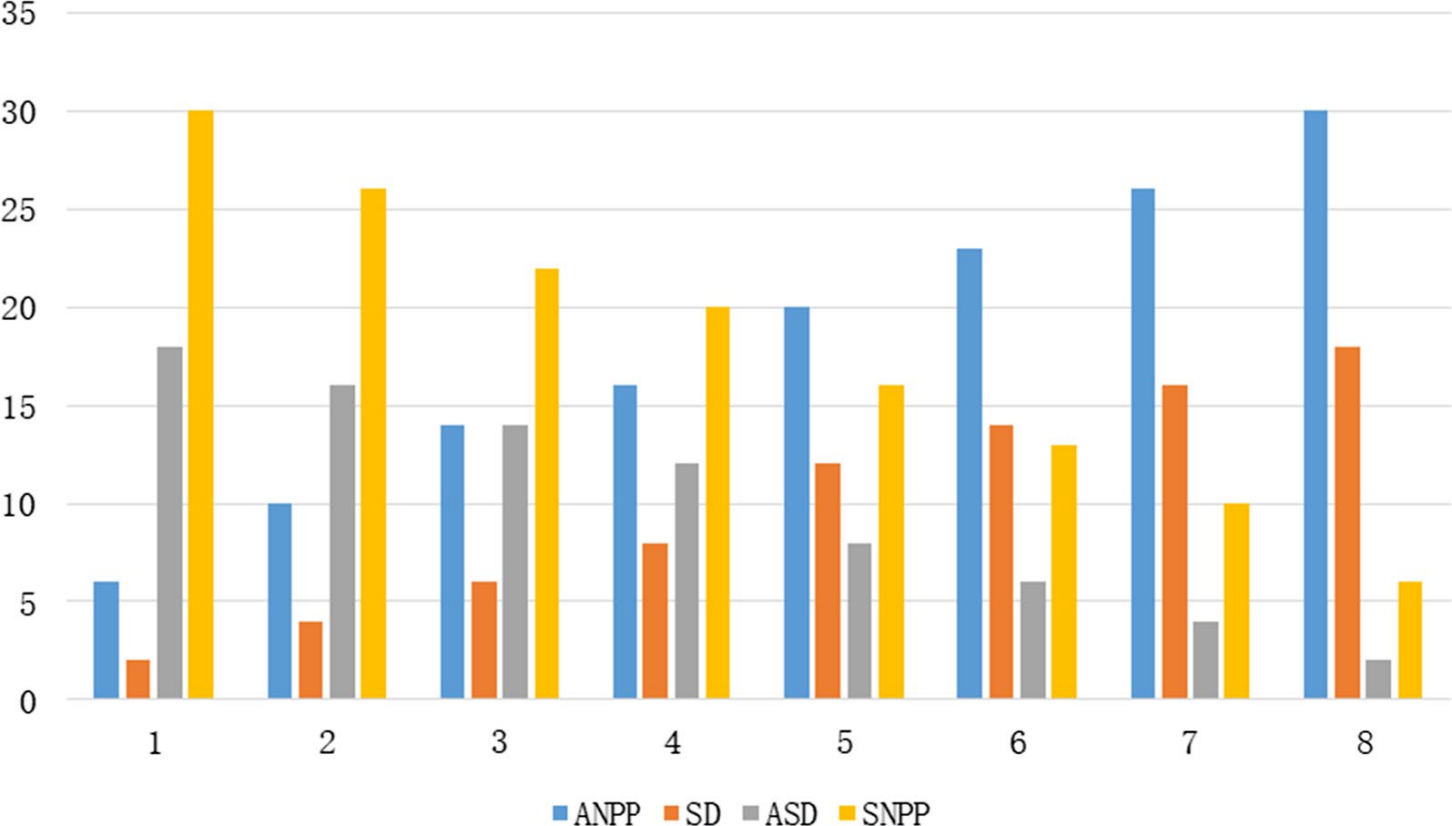
Patients of SD and ASD

Below, we analyze the minimum of the maximum working time corresponding to the change of values of relevant factors from an experimental perspective. In order to be more representative, the following cases are analyzed respectively: ET value increased from 2 to 8, DT value decreased from 8 to 2, SD value increased from 2 to 18, and ASD value decreased from 18 to 2. The test results are shown in Figs. 2 and 3.

Figure 2 shows the corresponding changes of ANPP when ET and DT change. As ET increases from 2 to 8, i.e. DT decreases from 8 to 2, SNPP decreases from 36 to 11. As ET time increases from 2 to 8, the time of examination service per patient gradually increases. While the free time of associate senior doctors is fixed, ANPP is also reduced from 36 to 11. Figure 3 shows the corresponding changes of ANPP and SNPP when SD and ASD change. As SD increased from 2 to 18, ASD decreased from 18 to 2, SNPP decreased from 30 to 6, and ANPP increased from 6 to 30. The free time of each senior associate doctor is fixed, and as ASD decreases from 18 to 2, the totally free time of all associate senior doctors gradually decreases. As a result, the number of patients referred by senior doctors gradually decreased. That the SNPP decreased from 30 to 6.

**Fig. 4:**
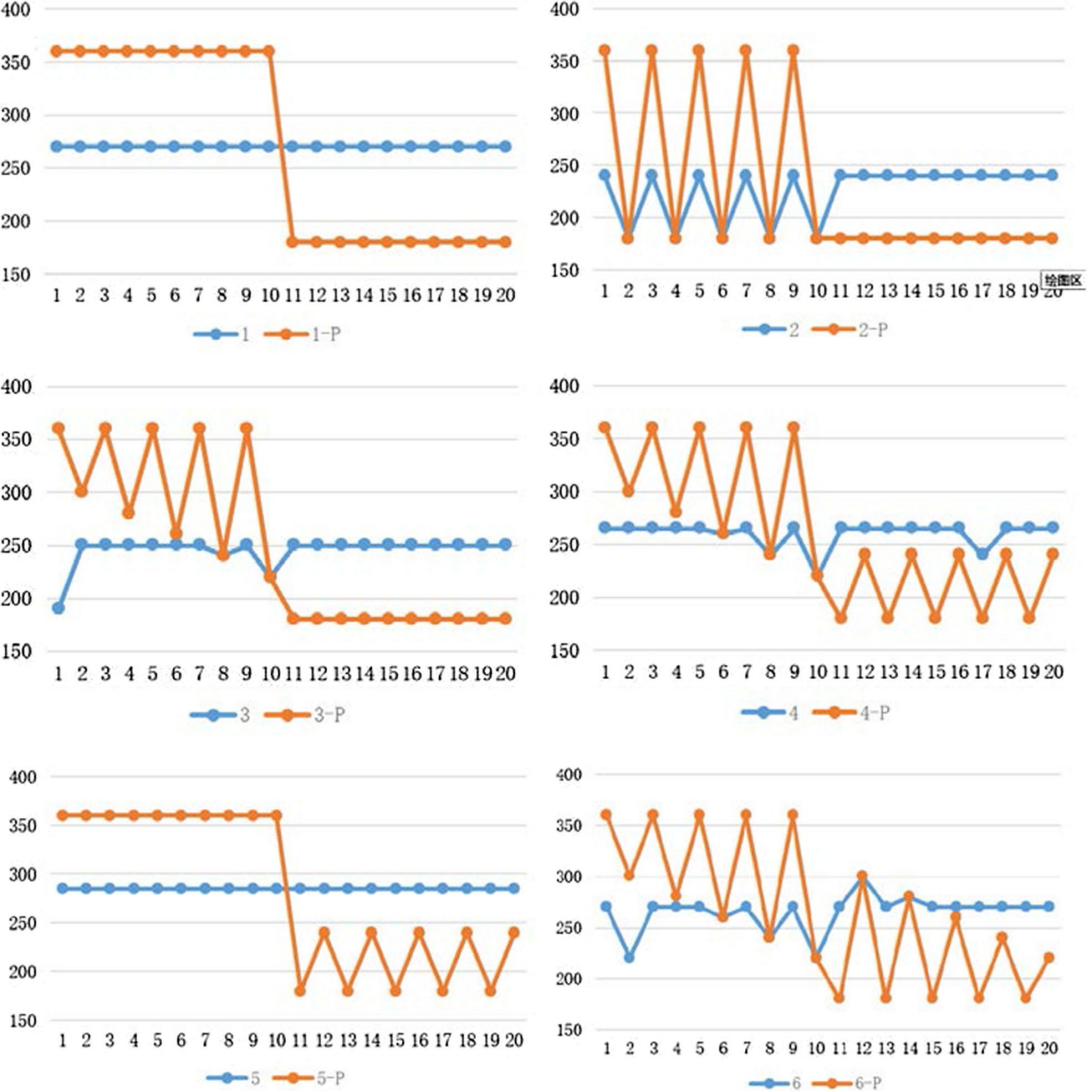
Minimum comparison of maximum working time

In Table 4, we give detailed experimental results before and after planning under the condition of whether senior doctors and associate senior doctors are busy or not. The parameters in this experiment are as follows: There are ten senior doctors and ten associate senior doctors respectively. Columns 3 through 12 in Table 4 represent the number of patients corresponding to senior doctors. The 13 to the 22nd cases are the number of patients corresponding to associate senior doctors; ET and DT time are 5 for each patient. Column 1 in Table 4 is the name, where 1 through 6 represent the six pre-planning scenarios.1-p to 6-p are the number of patients after the corresponding planning. In order to show the comparison before and after planning in more detail, the comparison of data trends before and after planning in 6 cases is shown in Fig. 4.

**Table 4:** Six detailed experimental results before and after planning

Name	LWT	Senior doctors										Associate senior doctors									
		1	2	3	4	5	6	7	8	9	10	11	12	13	14	15	16	17	18	19	20
1	360	36	36	36	36	36	36	36	36	36	36	18	18	18	18	18	18	18	18	18	18
1-P	270	− 18	− 18	− 18	− 18	− 18	− 18	− 18	− 18	− 18	− 18	18	18	18	18	18	18	18	18	18	18
2	360	36	18	36	18	36	18	36	18	36	18	18	18	18	18	18	18	18	18	18	18
2-P	240	− 24	0	− 24	0	− 24	0	− 24	0	− 24	0	12	12	12	12	12	12	12	12	12	12
3	360	36	30	36	28	36	26	36	24	36	22	18	18	18	18	18	18	18	18	18	18
3-P	250	− 34	− 10	− 22	− 6	− 22	− 2	− 22	0	− 22	0	14	14	14	14	14	14	14	14	14	14
4	360	36	30	36	28	36	26	36	24	36	22	18	24	18	24	18	24	18	24	18	24
4-P	265	− 19	− 7	− 19	− 3	− 19	0	− 19	0	− 19	0	17	5	17	5	17	5	12	5	17	5
5	360	36	36	36	36	36	36	36	36	36	36	18	24	18	24	18	24	18	24	18	24
5-P	285	− 15	− 15	− 15	− 15	− 15	− 15	− 15	− 15	− 15	− 15	21	9	21	9	21	9	21	9	21	9
6	360	36	30	36	28	36	26	36	24	36	22	18	30	18	28	18	26	18	24	18	22
6-p	280	− 18	− 16	− 18	− 2	− 18	0	− 18	0	− 18	0	18	0	18	0	18	2	18	6	18	10

In Fig. 4, the red line represents the working time of each doctor before the plan, and the blue line shows the working time of each doctor after the plan. The abscissa 1 to 10 represents 10 senior doctors, and 11 to 20 represents 10 associate senior doctors. Before planning, the working time of senior doctors are mostly higher than those of associate senior doctors. Senior doctors work longer time, while associate senior doctors work shorter time. After planning, the working time of senior doctors are significantly reduced, while those of associate senior doctors are increased, resulting in the working time of senior doctors and associate senior doctors being equal.

According to lean thinking, the patient outpatient service time is divided into examination service time, and diagnosis service time, and the problem is modeled as an integer programming problem to solve. As seen from the above experimental results, assigning patient examination services to associate senior doctors can effectively improve the work efficiency of senior doctors by 24% on average. At the same time, it also increased the number of patients served by associate senior doctors by an average of 50%. By using integer programming, the minimum maximum working time is obtained.

## Discussion

Outpatient service is the first step for hospital to provide high-quality and safe medical service for patients, and it is a key link to hospital quality control. The quality of outpatient procedures directly affects the image of the hospital, which reflects the overall service level of the hospital and relates to the overall interests of the hospital. Recent studies have shown that major problems in outpatient clinics include long patient waiting times, burnout among doctors, and the resulting inefficient use of resources.

In order to further improve patients’ medical experience and medical resource utilization, outpatient scheduling research has become a hot issue in medical operation management. This paper will discuss the latest progress in outpatient scheduling research from two aspects patient waiting time and patient flow. Then, the advantages and limitations of the mathematical programming model presented in this paper are discussed.

### Reduce waiting time

With the transformation of the medical service model and the development of the market economy, patients’ demand for medical services is getting higher and higher, and patient experience is getting more and more attention from medical circles. Good patient experience can improve patient treatment compliance, reduce medical costs, achieve better medical effects, and improve patient satisfaction. Long waiting time in the outpatient department has a great influence on timely access to medical services and medical experience. Many scholars have studied the problem of long waiting times.

Li et al. studied the satisfaction of outpatient service in community health centers and secondary and tertiary hospitals [20]. Patients at county and tertiary hospitals complained of long waiting times and low overall satisfaction with outpatient services. Compared with secondary hospitals, tertiary hospitals face greater challenges in outpatient satisfaction [21]. Several studies have shown that reducing uncanceled missed appointments can have a huge impact and improve resource utilization in physician productivity. Almuhaideb et al. predicted the situation of non-payment of outpatient appointments [22]. The Hoeffding tree algorithm is used to predict appointments with high absence risk in real-time. Then appropriate active interventions are set up to reduce the negative effects of absence. Barghash et al. studied the effect of predetermined overload percentage and patient interval on waiting for time, overtime and utilization rate [23]. The study found that over time increase excessively when both the basic interval and the overload percentage increase. If the basic interval must be reduced to achieve low overtime time, the utilization of doctors will also increase. Hribar et al. used discrete event simulation to model the outpatient eye clinic workflow to test a new scheduling template that could reduce patient waiting time and improve clinic efficiency [24]. Analyze EHR data and its compliance with templates to gain insight into new policies to better balance time to meet patient needs and minimize waiting time. Creps et al. used decision trees to analyze influencing factors significantly related to patients’ visiting behaviors, and then evaluated the possibility of patients not appearing [25]. On this basis, a dynamic appointment scheduler is developed, which uses different overbooking strategies for different appointment patients. Dynamic scheduler improves scheduling efficiency through overbooking.

Munavalli et al. proposed an overall patient scheduling model combining the status and information of all departments in the outpatient department for the long waiting time of patients [26]. This model guides the patient to the optimal path upon arrival and solves the problem of scheduling patients without appointments in real-time. Experimental results show that full patient scheduling significantly reduces waiting time, and real-time path optimization makes scheduling more efficient because it can immediately capture changes in the outpatient clinic. Berg et al. studied the use of constraint optimization modeling to balance doctors’ work schedules in specialized outpatient clinics, minimizing the variability of timetables, improving work efficiency, and thus reducing the maximum number of doctors making visits [27]. Nguyen et al. proposed precise and relaxing appointment scheduling rules to arrange the appointment of each new patient according to the uncertainty of future arrival [28]. Its scheduling rules are designed to maximize the use of program resources (i.e., physician staff) or, equivalently, to maximize the number of patients admitted. Numerical experiments test the proposed scheduling rules, and the experimental results show that the scheduling rules are efficient and effective in terms of service objective satisfaction and resource utilization. Silver et al. studied the reasons for the increased waiting time of cancer outpatient patients with large volumes [29]. Arranging too many patients in a short interval of clinic start time and arranging patients with more than the doctor's hourly patient capacity will increase the waiting time of patients. Doctors with the shortest waiting times for patients are interviewed and the best outpatient paths are identified. It proposes to reduce patient waiting time by benchmarking the optimal outpatient pathway. Which records and analyzes patient workflow and scheduling processes.

The above methods can improve patients’ medical examination and patient satisfaction by reducing patients’ waiting time. In this paper, a mathematical programming model combined with lean thinking is proposed to effectively reduce the waiting time of patients through a two-stage outpatient process. In addition, on the premise of not changing the outpatient process, this method plans the implementation space and doctors of the outpatient process from the perspective of space, and increases the patient flow while improving the patient attendance rate of associate senior doctors. Hospital management can further improve the service management model of the hospital by using lean thinking for reference. Eliminating redundant links and patients’ unnecessary waiting in hospital management, and developing a standardized optimization flow chart of the hospital treatment process. Healthcare reform needs to do more to reduce patient waiting time, such as improving the quality of primary care diagnosis and treatment, and creating more targeted referral systems.

### The patient flow

Many scholars have studied the refined management method of patient flow to increase the capacity of outpatient reception and the number of inpatients by effectively increasing mobility.

Lin et al. proposed a novel heuristic algorithm based on two-stage simulation to evaluate and optimize various tactical and operational decisions for multiple objectives [30]. Resource allocation plans are sought in the first phase, and overall outpatient appointment schedules are determined based on patient categories and daily operational level service discipline. Hahn et al. proposed outpatient dynamic template scheduling, a new technology combining active optimization and online optimization, and applied it to the scheduling problem of chemotherapy outpatient clinics [31]. A deterministic optimization model and appointment samples are used to create an active template for the expected dates of chemotherapeutic centers, which solves the dynamic uncertainty caused by appointment requests arriving in real-time and the uncertainty caused by last-minute schedule changes. Scheduling an outpatient appointment involves a complex set of factors and different stakeholders. Families, administrators, and clinicians may have different experiences in scheduling clinic appointments. Maira et al. studied the views and experiences of scheduling pediatric outpatient appointments from the perspective of stakeholders [32]. Qualitative content analysis is used to analyze the three stakeholder groups. It is found that the treatment process, skills, and services have the greatest impact on the pediatric outpatient scheduling system.

DeWaters et al. reviewed the commissioning methods for internal medicine residents [33]. The effects of block and traditional outpatient scheduling on inpatient-related outcomes are systematically examined. In the lastest decade, nearly half of internal medicine residents have implemented block-based outpatient scheduling. While block-based scheduling has improved residents’ satisfaction, the conflict between inpatient and outpatient responsibilities, and outpatient training time, there may be important trade-offs with poor continuity of care. Klassen et al. investigated the circumstances under which additional mid-level service providers (MLSP) could be added to a single-physician clinic and identified scheduling strategies in a single-phase environment [34]. Compared with a single-phase system in which the physician performs all parts of the service, the results showed that the addition of MLSP reduced patient waiting time, patient flow time, and physician service time. Steen et al. adjusted the location of workload generation and activities in working days, improving clinic utilization rate and a total number of visits in the clinical pharmacy expert compound group [35]. The implementation of the intervention shows that pharmaceutical administrators can improve workload and obtain quality care by cooperating with clinical pharmaceutical experts.

Munavalli et al. modeled the outpatient clinic as a multi-agent system, and proposed an intelligent real-time scheduler that could schedule patients and resources according to the actual state of the department [36]. The results show that the intelligent real-time scheduler significantly improves the performance indexes such as waiting time, cycle time, and utilization rate. The outpatient system becomes a pull system by scheduling resources and patients according to system status and demand. The online reservation scheduling system is designed to solve the problem of the traditional reservation scheduling systems. In Iran, most outpatient clinics and our study population are facing high rates of unattended patients and long waiting times due to the non-use of an online appointment systems. Habibi et al. studied the effect of online appointment scheduling systems by comparing the evaluation indicators of appointment scheduling before and after intervention [37]. This pre- and post-pilot study is conducted in 10 outpatient clinics in different specialties. An online appointment scheduling system effectively reduced patient wait time, and patient absence rate and improved physician punctuality.

The above methods reduce patient waiting time and improve the utilization of doctors and medical resources by increasing mobility. The mathematical programming model presented in this paper improves the work efficiency of senior doctors, and then gives senior doctors more time to provide services to patients in greater need. In addition, it also improves the number of associate senior doctors’ patients. On the one hand, it effectively alleviates the waiting time of patients and accumulates diagnosis and treatment experience in difficult and miscellaneous diseases, which is conducive to the rapid growth of associate senior doctors. Medical care has become one of the largest industries in the world. In recent years, the cost of medical care in developed countries has risen sharply, of which about one-third is from hospital expenses. Health and government departments of various countries have issued relevant policies, forcing hospitals to improve operating efficiency, control costs, and increase income. The improvement of patient flow is in essence to improve the utilization rate of medical resources, reduce the average hospitalization cost per patient, and at the same time, it is more conducive to the hospital to control costs and increase income.

### Advantages and limitations

We actively involve hospital management and medical staff constructing of the mathematical model, to more comprehensively consider the complexity of the outpatient scheduling model, which increases the possibility of model planning results to guide decision-making, and effectively solves the challenging problems in analysis, statistics, and modeling methods. The mathematical model is based on constraint relations, and the minimum and maximum working time is solved automatically by CPLEX. The mathematical model is easy to model and solve. By providing a set of relevant input parameters to the model, relevant information about senior physicians, senior associate physicians, and patient distribution can be solved.

Based on the existing constraint relations of the mathematical model proposed in this paper, other constraint relations can be added to the model. For example, the restrictions on the working time of individual doctors, doctors’ and patients’ preferences, and the capacity of relevant medical resources. This mathematical model is customized according to outpatient procedures, but it can also be applied to other spatial planning problems. This mathematical model is constructed after discussion with outpatient management and can only be applied to guide the allocation of doctors and patients when patient flow, doctors, or related medical resources change.

## Conclusion

Aiming at the key problems in outpatient service, this paper considers the work efficiency of senior doctors and the waiting time of patients, and combines lean thinking and integer programming method to optimize outpatient service. This study enriches and improves the theory and method system of medical operation management, especially outpatient service scheduling. This method has strong practicability and can provide a theoretical basis and decision support for hospitals to the perfect outpatient service systems and reasonably schedule outpatient resources. It can help the hospital reduce the cost of outpatient operations, improve the utilization rate of medical resources and patient satisfaction, and comprehensively improve the quality of medical service.

In this paper, based on lean thinking, the outpatient service is subdivided into examination service and diagnosis service, and the examination service provided by the senior doctor is assigned to the senior associate doctor. For senior doctors, energy consumption and working time are reduced, so that more energy and time are available to provide services to other patients in need; For patients, the waiting time of patients is reduced without affecting their medical treatment; For the associate senior doctors, the number of patients received has significantly increased, which not only helps to improve the professional level but also helps to solve the problem of difficult medical treatment. Using CPLEX to solve the outpatient planning problem, the minimum and maximum working time are obtained, and the examination service and diagnosis service allocation are planned from a fine perspective. Compared with those without outpatient service planning, the work efficiency of senior doctors increased by 25% on average, and the patient flow of associate senior doctors increased by 50% on average.

## Data Availability

All code is open source with no restrictions and is available from https://github.com/hometownjlu/outpat-cap-plan.
